# Traumatic stifle injury in 72 cats: a multicentre retrospective study

**DOI:** 10.1177/1098612X211028834

**Published:** 2021-07-13

**Authors:** Mario Coppola, Smita Das, George Matthews, Matteo Cantatore, Luis Silva, Pilar Lafuente, Elvin Kulendra, Hannah Clarke, Jessica McCarthy, Nuria Fernandez-Salesa, Sorrel Langley-Hobbs, Josep Aisa, Timothy Parkin, Elena S Addison

**Affiliations:** 1 Small Animal Hospital, University of Glasgow, Glasgow, UK; 2 Davies Veterinary Specialists, Higham Gobion, UK; 3 Queen Mother Hospital for Animals, Royal Veterinary College, Hatfield, UK; 4 Anderson Moores Veterinary Specialists, Hursley, UK; 5 Department of Animal Medicine and Surgery, Universidad CEU Cardenal Herrera, Valencia, Spain; 6 The Royal (Dick) School of Veterinary Studies, Roslin, UK; 7 Hospital Veterinario UCV, Universidad Catolica de Valencia, Valencia, Spain; 8 University of Bristol, Langford Veterinary Services, Bristol, UK; 9 University of Tennessee College of Veterinary Medicine, Knoxville, TN, USA

**Keywords:** Stifle, luxation, disruption, traumatic injury

## Abstract

**Objectives:**

The aim of the study was to describe traumatic stifle injury in cats and report complications and long-term outcome.

**Methods:**

The medical records from seven veterinary hospitals of cats treated for traumatic stifle injury were reviewed. Long-term follow-up data were collected from referring veterinarians and using the Feline Musculoskeletal Pain Index, collected from owners.

**Results:**

Seventy-two cats were included in the study. The most common combination of ligament injury involved both cruciate ligaments and the lateral collateral ligament (25.4%). Medial meniscal injury was more common (66.2%) than lateral meniscal injury (59.4%). A temporary transarticular pin was used intraoperatively to aid reduction in 23/73 (31.5%) surgeries. Postoperative immobilisation was applied in 41/72 (56.9%) cats with a mean duration of 4.8 weeks. Short-term complications occurred in 40/64 (62.5%) cats. Long-term complications occurred in seven (17.5%) cats. Overall outcome was excellent in 25/61 (41%) cats, good in 13/61 (21.3%) cats, fair in 11/61 (18%) cats and poor in 12/61 (19.7%) cats. Mean length of follow-up was 29.6 months (range 0.5–204). A significantly poorer outcome was observed in cats with medial meniscal injury and those undergoing revision surgery. Use of a transarticular pin when left in situ for postoperative immobilisation was associated with a poorer outcome (P = 0.043) and a higher risk of complications (P = 0.018). Postoperative immobilisation was not related to outcome.

**Conclusions and relevance:**

Traumatic stifle injury in cats can lead to rupture of multiple ligaments causing significant instability of the joint. Surgical treatment is associated with a high rate of short-term complications, although long-term outcome may still be good to excellent in the majority of cats (62.3%). In cats where follow-up was available, postoperative immobilisation had no positive effect on outcome and may not be required. Leaving a transarticular pin for postoperative immobilisation is not recommended as it was significantly associated with a poorer outcome and a higher complication rate.

## Introduction

Stifle joint disruption, derangement or luxation is relatively uncommon in cats.^1^ These terms are used interchangeably. Severe instability of the stifle is due to damage of the primary joint restraints, such as cruciate and collateral ligaments and secondary joint restraint tissues, such as the joint capsule, menisci, tendons and muscles.^2^ These injuries are often caused by high-energy trauma such as road traffic accidents, falls or catching the limb while jumping a fence.^3,4^

Typically, the medial collateral ligament is more commonly affected than the lateral collateral ligament.^5^ The most common combination of injuries reported in the literature involves the cranial and caudal cruciate ligaments and the medial collateral ligament.^6,7^

The treatment goals following stifle disruption are to limit further damage to the articular surfaces and remaining supporting structures, restore joint stability and alignment, and maintain a normal range of motion.^8^

In cats, surgical stabilisation of a cranial cruciate ligament injury is usually achieved with a lateral fabello-tibial suture, while stabilisation of a caudal cruciate ligament injury can be accomplished via a fibula-patellar suture.^9^ However, surgical stabilisation of the caudal cruciate ligament may not be essential in all cats.^6^ Described techniques for the surgical management of collateral ligament injury include primary repair, replacement of the damaged ligament with autogenous tissue or suture material, or a combination of both.^8^ A temporary transarticular pin can be used intraoperatively to facilitate maintenance of reduction and appropriate tensioning of prosthetic sutures.^2^

Typically, the joint is immobilised following surgery to provide adjunctive joint stabilisation during healing and protection of the primary repair. However, immobilisation has several negative effects on the joint such as decreased synovial fluid production, reduction of cartilage stiffness and thickness, decreased range of motion and development of degenerative joint disease; prolonged immobilisation also causes loss of muscle mass.^8^ Therefore, its use and duration postoperatively remain controversial. Immobilisation can be achieved using trans-articular pinning, transarticular external skeletal fixation or external coaptation,^10,11^ although the latter is more difficult to maintain in place and it can lead to severe bandage-related injuries. Alternatively, in order to maintain some joint motion and reduce the deleterious effects of immobilisation, a hinged transarticular external skeletal fixator can be used.^8^

Complications associated with the surgical treatment of stifle disruption are numerous and include the following: pin loosening, bending or breakage; femoral or tibial fracture through pin holes; pin tract discharge; infection; a loss of range of motion of the joint and persistent instability; lameness; and degenerative joint disease.^12^ The latter is thought to be due more to the severe joint injury than to complications of surgical treatment. Despite this, outcomes are reported to be satisfactory to good in the majority of cats.^9^ However, only small case series have been reported in the literature, with no objective outcome measures or clarification of what a ‘satisfactory to good’ outcome means.

A large cohort of cats with traumatic stifle injury has not been previously described in the literature. The purpose of this paper is to describe traumatic stifle injury in a large number of cats and to investigate factors associated with outcome and complications. We hypothesised that a lack of postoperative immobilisation would be associated with a poorer outcome.

## Materials and methods

Medical records from seven referral hospitals in the United Kingdom were reviewed (Small Animal Hospital, University of Glasgow; Queen Mother Hospital for Animals, Royal Veterinary College; Davies Veterinary Specialists; Anderson Moores Veterinary Specialists; Hospital for Small Animals, The Royal [Dick] School of Veterinary Studies; Langford Veterinary Small Animal Hospital; and North Downs Specialist Referrals) from October 2008 to June 2018 and included client-owned cats treated for traumatic stifle injury. Ethical approval for this study was granted by the institutional animal research ethics committee of the University of Glasgow, School of Veterinary Medicine, Glasgow, UK (Ref03a/18).

Data collected from the medical records included breed, sex, age, affected limb, cause of injury, presence of concurrent injuries, clinical findings, preoperative, immediate postoperative and follow-up radiographic findings, intraoperative findings, injury configuration, surgical procedures performed, use of postoperative immobilisation, type and duration of immobilisation, revision surgery required, survival to discharge, duration of hospitalisation, short- and long-term complications, and overall outcome. Cats presenting with a traumatic stifle injury were included in the study. Animals in which the only ligament injured was the cranial cruciate ligament and animals with simultaneous bilateral stifle luxation were excluded from the study. This selection was necessary owing to the difficulty in differentiating acute traumatic injury from degenerative cranial cruciate disease due to the retrospective nature of the study, and the possibility that unwitnessed trauma may have occurred in cats with an outdoor lifestyle.

A definitive diagnosis was established on the basis of clinical, radiographic and intraoperative findings. Evidence for ligament damage was based on the presence of partial or complete disruption of the substance of the ligament or ligament–bone avulsion.

All cats were treated surgically. The affected joints were explored to determine the presence of cruciate (cranial and caudal) ligament, meniscal or collateral ligament tears. Cruciate ligament tears were classified as complete or partial. Collateral ligament injuries were graded as follows: grade I in case of parenchymal haematoma/oedema (only few fibres torn); grade II in case of partial tear of the ligament; and grade III in case of complete ligament rupture rupture.^13^ Injuries were addressed at the discretion of the operating surgeon and performed surgical techniques were recorded. Partial or complete meniscectomy was performed when meniscal damage was encountered.

The occurrence and nature of postoperative complications were recorded. For the purpose of this study, complications were categorised as major (surgical intervention required) or minor (managed non-surgically). Details of any revision surgery were recorded. Furthermore, complications were classified as short- (STCs) or long-term complications (LTCs), if reported before or after 8 weeks from the initial surgery, respectively.

Long-term follow-up was obtained from referring veterinarians following owners’ consent. The Feline Musculoskeletal Pain Index (FMPI) questionnaire version 10, 2015 (https://journals.plos.org/plosone/article/file?type=supplementary&id=info:doi/10.1371/journal.pone.0131839.s001) was sent by post to all owners of cats that were still alive at the time of the study, where the full owner address was available. The total FMPI score and percentage possible score (FMPI%poss) were calculated. Calculation of the FMPI%poss was performed by taking the total score for the cat and dividing by the total possible points (the number of questions answered multiplied by four). Higher totals indicated less impairment with a possible range of 0–16 (eg, a FMPI%poss score of 16 was considered to exhibit no deficit in function).

The overall outcome was determined from the FMPI score where available or from the latest follow-up obtained from referring veterinarians’ records. The overall outcome was assigned into one of the following categories: excellent (return to full function without lameness); good (occasional/intermittent mild lameness); fair (persistent mild/moderate lameness); and poor (moderate/severe lameness, amputation or euthanasia).

### Statistics

All collected information was recorded on an Excel spreadsheet (Microsoft Excel for Office 365). Univariable, followed by multivariable, logistic regression analysis was used to identify risk factors for ‘poorer outcomes’ and ‘complications’. All potential risk factors were examined individually to identify potential association with each outcome. Variables with an initial *P* value of <0.25 were considered for inclusion in final multivariable models. Continuous variables were examined as categorical variables, where possible, in order to identify the best fit in each model. Categorical variables were collapsed when individual levels of that variable were not significantly different to the reference level, and when it made biological sense to do so. In other words, remembering that it is important to have as few parameters in a model as possible, a variable with four levels may have been reduced to two or three levels when there was no evidence of a statistical difference between the levels that were combined. Variables were entered into multivariable models based on the size of the *P* value and univariable odds ratio (OR). Models were built in a manual stepwise process until the addition of no further significant variables improved the overall fit of the model. In both models, all remaining variables were entered, one at a time, to examine any effects of confounding. Confounding was considered to be present when the ORs of retained risk factors was modified by more than 30%.^14^

## Results

### Signalment

Seventy-two cats were included in the study. One cat suffered traumatic stifle injury in both limbs at different times during the study period and was therefore included twice. Data for 73 stifles were evaluated. Forty cats (54.8%) were male (all neutered) and 33 (45.2%) were female (30 neutered; three entire). Sixty-three cats (86.3%) were domestic short- or longhair, with 10 cats (13.7%) represented by other pedigree breeds. The median age was 8 years (range 9 months to 12 years, 6 months).

### Clinical findings

The left side was affected in 50.7% (n = 36/71) of cats, while the right side was affected in the remaining 49.3% (n = 35/71) cats. The cause of the trauma was unknown in 68.5% (n = 50/73) of cats and known in 31.5% (n = 23/73) of cats. The type of trauma included road traffic accidents (n = 16), having the limb caught in a fence (n = 5), cat fight (n = 1) and falling from a height (n = 1).

Twenty-two cats (30.1%) had concurrent trauma with the following injuries: hip luxation (n = 5); sacroiliac luxation (n = 5); pelvic fractures (n = 4); skin laceration (n = 4); pulmonary contusion (n = 3); tarsocrural joint luxation/instability of the ipsilateral limb (n = 3); femoral fracture of the ipsilateral limb (n = 2); tibial fracture of the ipsilateral limb (n = 2); degloving skin injury of the ipsilateral limb (n = 2); calcaneoquartal joint disruption of the ipsilateral limb (n = 1); radial nerve neuropraxia of the ipsilateral limb (n = 1); fibular fracture of the ipsilateral limb (n = 1); tension pneumothorax (n = 1); tarsal fracture (affected bone not specified) of the ipsilateral limb (n = 1); retroperitoneal haematoma (n = 1); caudal cruciate ligament rupture in the contralateral limb (n = 1); and abdominal wall rupture (n = 1).

### Surgical findings

The cranial cruciate ligament was ruptured in 87.3% of cats (n = 62/71) of which 87.3% (n = 54/62) were complete and 6.5% (n = 4/62) were partially ruptured; for the remaining four cats the type of lesion was not specified. The caudal cruciate ligament was ruptured in 77.5% of cats (n = 55/71) of which 81.8% (n = 45/55) were complete and 16.4% (9/55) were partially ruptured; in one cat the type of lesion was not specified. The medial collateral ligament was injured in 53.5% of the cats (n = 38/71), of which 42.9% (n = 15/35) were a grade II sprain and 57.1% (n = 20/35) were completely ruptured (grade III). The lateral collateral ligament was injured in 69% of the cats (n = 49/71), of which 53.1% (n = 26/49) were a grade II sprain and 32.7% (n = 16/49) were completely ruptured (grade III); for the remaining seven cats the type of lesion was not specified.

**Figure 1:**
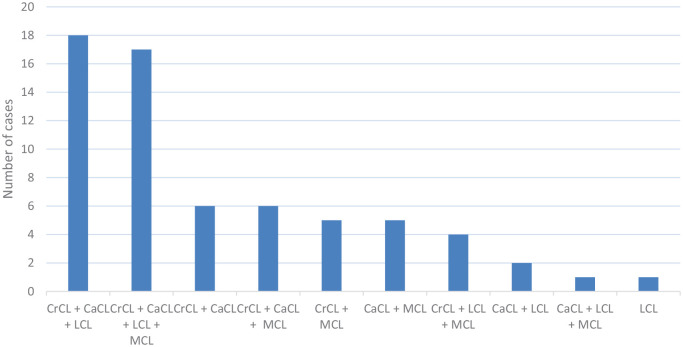
Combination of ligament injuries. CrCL = cranial cruciate ligament; CaCL = caudal cruciate ligament; LCL = lateral collateral ligament; MCL = medial collateral ligament

All the different combinations are illustrated in Figure 1. Twelve different combinations of ligament injuries were reported; the most frequent combination encountered was damage of the lateral collateral ligament and of both cruciate ligaments, reported in 25.4% (n = 18/71) of patients.

The medial meniscus was damaged in 66.2% (n = 43/65) of the cats and the lateral meniscus was damaged in 59.4% (n = 38/64) of the cats. In 50% (n = 32/64) of the cats with lateral meniscus injury, the medial meniscus was also injured.

### Surgical techniques

Sixty-seven percent (n = 47/70) of the procedures were performed by a diplomate as primary surgeon. Mean surgical time was 120.7 mins (range 45–330).

The ligament and meniscal injuries were addressed at the discretion of the surgeon, depending on surgeon choice and type of lesions. Sixty-two cats had a ruptured cranial cruciate ligament, which was treated using a fabello-tibial suture in 58 cats, with nylon in 23 cats, fibre wire in nine cats, polydioxanone in six cats, polyester suture in three cats, polypropylene in three cats and, for 14 cats, the material used was not specified. In the remaining four cats with a cranial cruciate ligament injury, the technique used was not specified.

Fifty-five cats had caudal cruciate ligament injury, which was addressed in 14 cats with a fibulo-patellar suture. In the remaining cats the caudal cruciate ligament injury was not addressed.

Twenty cats had complete rupture (grade III sprain) of the medial collateral ligament, and in 17 of these a prosthetic ligament was used. Prosthetic ligament materials included polyester (four cats), nylon (four cats), polydioxanone (four cats), polypropylene (two cats) and orthopaedic wire (one cat); the material used was not specified in two cats. The sutures were placed with the aid of orthopaedic screws (13 cats), bone tunnels (three cats) and suture anchors (one cat). In two cats the ligament injury was not addressed, and in one cat primary repair of the ligament was attempted. Fifteen cats were diagnosed with a grade II sprain. The injury was not addressed in eight cats, in five cats a prosthetic ligament was applied, in one cat the ligament was primarily repaired and in one cat the technique used was not specified.

Sixteen cats had complete rupture (grade III sprain) of the lateral collateral ligament, and in nine of these a prosthetic ligament was used. Prosthetic ligament materials included nylon (four cats), polydioxanone (one cat), polyester (one cat), polyethylene (one cat) and in one cat a fascia lata graft was used; the material used was not specified in one cat. The sutures were placed with the aid of orthopaedic screws (four cats), suture anchors (three cats), and a combination of orthopaedic screw and bone tunnel (one cat); in one cat it was not specified how the suture was anchored. In five cats the ligament injury was not addressed and in two cats primary repair of the ligament was attempted. Twenty-six cats were diagnosed with a grade II sprain of the lateral collateral ligament. In nine cats a prosthetic ligament was applied; in 13 cats the injury was not addressed surgically; in three cats the ligament was primarily repaired and in one cat the surgical technique used was not specified.

A transarticular pin for temporary intraoperative joint stabilisation was used in 31.5% (n = 23/73) of cats. Of these, the transarticular pin remained in situ for 4–7 weeks (mean 4.6) in 9.6% (n = 7/73) of cats, to provide postoperative immobilisation.

Postoperative joint immobilisation was used in 56.9% (n = 41/72) of patients. A transarticular external skeletal fixator was used in 33 cats (80.5%), a transarticular pin alone in five cats (12.2%), a transarticular pin combined with a transarticular external skeletal fixator in two cats (4.9%) and a splinted bandage was applied in one cat (2.4%). Mean duration of postoperative immobilisation was 4.8 weeks (range 0.5–12). One of the cats with transarticular external fixator suffered from femoral fracture 3 days postoperatively. Therefore, the external fixator was removed at 0.5 weeks.

All patients survived to discharge, with a mean duration of hospitalisation of 3.8 days (range 1–12).

### Complications

Intraoperative complications occurred in 8.2% (n = 6/73) of cats. One cat suffered intraoperative hypotension; one cat experienced failure of the over the top graft technique to address cranial cruciate ligament damage due to excessive tension during tightening, so an extracapsular suture technique was used instead; one cat developed lateral patellar luxation, which was noted immediately after surgery and the patient underwent a second surgery under the same anaesthetic to address the patellar luxation; one patient developed a fissure in the proximal femur following insertion of the most proximal pin of the external skeletal fixator; one cat exhibited excessive reduction in flexion following stabilisation, presumably due to excessive tightening of the extracapsular suture used for stabilisation; and in one cat there was a technical error in the placement of a surgical screw in the fibular head for repair of a lateral collateral ligament (the technical error was not described).

STCs (<8 weeks) occurred in 62.5% (n = 40/64) of patients, of which 50% (n = 20/40) were classified as major and the remaining 50% (n = 20/40) as minor. All the complications that led to amputation were classified as major, but amputations were not counted as revision surgeries. Limb amputation was performed in six cats. Complications that led to such limb amputation included bone fractures in four cats, stifle re-luxation in one cat and extended skin necrosis in one cat. Included STCs recorded were reduced range of motion of the operated stifle (n = 8/40), recurrence of stifle luxation (n = 5/40), persistent severe lameness (n = 5/40), pin tract infection (n = 4/40), femoral fracture (n = 4/40: all fractures occurred at external fixator pin sites), lateral patellar luxation (n = 2/40), medial patellar luxation (n = 2/40), transarticular pin migration (n = 2/40), broken transarticular pin (n = 2/40), transarticular pin loosening (n = 1/40), sciatic neuropraxia (n = 1/40), peroneal neuropraxia (n = 1/40), skin necrosis (n = 1/40), marked quadriceps atrophy (n = 1/40) and loosening of the screw placed in medial proximal tibia for medial collateral ligament prosthesis (n = 1/40). Of the STCs, sciatic neuropraxia, peroneal neuropraxia and marked quadriceps atrophy (n = 3/40) were classified as minor, while all the other STCs reported (n = 37/40) were classified as major.

LTCs (⩾8 weeks) were reported in 17.5% (n = 7/40) of cats, of which 57.1% (n = 4/7) were classified as minor and the remaining 42.9% (n = 3/7) as major. Reported LTCs were persistent lameness (n = 3/7), stifle instability following transarticular external skeletal fixator removal (n = 1/7), pain and reluctance to move the stifle (n =1/7), recurrence of stifle luxation (n = 1/7) and screw loosening (n = 1/7). Of the reported LTCs, 5/7 were classified as minor (persistent lameness, pain and reluctance to move the stifle, and stifle instability following transarticular external skeletal fixator removal) and 2/7 as major (recurrence of stifle luxation and screw loosening).

The overall complication rate was 62.3% (n = 43/69) as four cats reported both STCs and LTCs.

Revision surgery was performed in 23.9% (n = 17/71) of cats. Reasons for revision included patellar luxation (n = 3/17 [17.6%]), recurrence of stifle luxation (n = 2/17 [11.8%]), implant failure (n = 2/17 [11.8%]), femoral fracture (n = 2/17 [11.8%]), tibial fracture (n = 1/17 [5.9%]), breakage of the intra-articular pin (n = 1/17 [5.9%]) and pin migration (n = 1/17 [5.9%]). In five cats (29.4%), the complication and reason for revision were not specified.

The use of a transarticular pin left in situ for postoperative immobilisation was associated with a higher rate of complications (OR 4.35, *P* = 0.018). Pin loosening occurred in three cats, pin migration in two cats and the pin broke in two cats. When the transarticular pin was only used intraoperatively for temporary stabilisation, it was not related to a higher complication rate (*P* = 0.324). No other factors were identified as being associated with the development of complications.

### Outcome

Mean follow-up time was 29 months and 2 weeks (range 2 weeks to 204 months). Clinical outcome at the time of last follow-up was excellent in 41% (n = 25/61) of cats, good in 21.3% (n = 13/61), fair in 18% (n = 11/61) and poor in 19.7% (n = 12/61). For statistical analysis, outcome was grouped together as either an ‘excellent/good’ outcome or a ‘fair/poor’ outcome. Factors associated with a poorer outcome were revision surgery (OR 14.2, *P* = 0.00), lack of cranial cruciate ligament injury (OR 6.44, *P* = 0.049), presence of medial meniscal injury (OR 5.5, *P* = 0.015) and the use of a transarticular pin left in situ for postoperative immobilisation (OR 3.7, *P* = 0.043). A lack of postoperative immobilisation was not related with poorer outcome. FMPI questionnaires were sent to 11 owners, of whom eight completed them. The average time between the surgery and completion of the questionnaire was 5.6 years. Four cats had a FMPI%poss score of 16, consistent with no functional deficits. The other four cats had FMPI%poss scores of 14.1, 13.4, 13.1 and 12.5, respectively. Mean FMPI% score was 14.7 and mean age at the time of questionnaire completion was 11.8 years.

## Discussion

Traumatic stifle injury in cats is an uncommon injury usually associated with the rupture of multiple ligaments causing significant instability of the joint. In many cats the traumatic event is unknown, which is likely due to the outdoor independent lifestyle of many cats. Concurrent injuries were reported in 30% of cats, but the presence of these injuries was not found to be related to complications or outcome. The most common combination of injuries in the present series was damage to both cruciate ligaments and the lateral collateral ligament, followed by injury of both cruciate and collateral ligaments. This is in contrast to previous reports where the most frequent combination of injuries was rupture of both cruciate ligaments, and the medial collateral ligament.^2,10,15^ In our study, this combination was only reported in 8.5% of cats. It is likely that our results are more representative of the feline population in the UK because we analysed a much larger number of cats than previous studies. In cats with a known cause, there was often substantial, high-energy trauma to the joint; therefore, injury of all four ligaments is understandable. However, there is also evidence that the lateral collateral ligament was damaged more frequently than the medial overall. The medial collateral ligament has a greater area of insertion on the medial aspect of the tibia, in comparison with the lateral collateral ligament, which inserts on the fibular head. This anatomical difference could explain why the lateral collateral ligament was more frequently damaged than the medial collateral ligament. The medial collateral ligament insertion may be more robust, reducing the likelihood of rupture with traumatic injury to the stifle joint.

The medial meniscus was more frequently damaged than the lateral meniscus in our study, and the caudal pole was the region more frequently damaged. A review on feline stifle anatomy demonstrated several anatomical differences between the lateral and medial menisci. There are two ligaments that attach each meniscus to the tibia (meniscotibial ligaments) and one that attaches the lateral meniscus to the femur (meniscofemoral ligament).^16^ It is therefore less likely to be injured than the relatively immobile medial meniscus and that could explain the findings of our study.

In our study, medial meniscal injury was associated with a poorer outcome. We assumed this was likely due to the development of osteoarthritis and articular cartilage damage following partial or complete meniscectomy, as has been shown in previous studies.^17^ In dogs, compromised function of the meniscus by either medial meniscal release or medial complete meniscectomy results in stress concentration, which may predispose to osteo-arthritis.^18^ Another study revealed that performing meniscal repair instead of partial meniscectomy in dogs with select meniscal tears may mitigate the development of degenerative joint disease.^19^ To our knowledge, meniscal repair has not been reported in cats. Future ex vivo and clinical studies should aim to refine the treatment of specific meniscal injuries in cats.

A lack of injury to the cranial cruciate ligament was also associated with a poorer outcome. Clinically, it is difficult to see why this would be the case. There were only nine cats without cranial cruciate ligament injury. These patients’ data were reviewed looking for a statistically significant correlation, but none was found. However, eight cats had a caudal cruciate ligament injury alongside a collateral ligament injury. Only two of these cats had the caudal cruciate ligament instability surgically addressed. In the remaining cats, they were either managed with surgical prosthetics for the collateral ligaments alone or a transarticular external skeletal fixator was placed. In four of these cats, persistent caudal drawer was reported on re-examination, which could be related to the poorer outcome. Treatment of the caudal cruciate ligament is controversial, but these results may suggest that in the presence of an intact cranial cruciate ligament, a patellofibular suture should be placed to prevent persistent caudal tibial subluxation. The sample size was very small, however, and no firm conclusions could be made, but this factor may warrant further investigation.

Postoperative immobilisation after traumatic stifle injury in cats is controversial. In our study, when all postoperative immobilisation techniques were grouped together and analysed with multivariate logistic regression, no effect on either outcome or complication rate was identified. However, when the transarticular pin group was assessed separately, the use of a transarticular pin left in situ for postoperative immobilisation was related to a higher complication rate and a poorer outcome. Transarticular pinning as a sole method for stabilising luxated stifles in cats was first recommended by Hoffman et al in 1985,^20^ with the aim of maintaining joint reduction and stability enabling fibrosis of the periarticular soft tissues. The disadvantages of this technique include implant failure through pin loosening, migration and bending.^15^ For effective immobilisation the pin needs to be relatively large, which could damage the surrounding structures within the stifle joint.

In our study, a transarticular pin was used in 23 cats to maintain stifle reduction during surgery, and in seven of these patients it was left in situ for postoperative immobilisation. The size of the transarticular pin used was only recorded in five cats, varying from 1.2 mm to 2 mm. Damage to cartilage and intra-articular structures caused by a large pin may have contributed to the poorer outcome due to greater degenerative joint disease. In contrast, when the transarticular pin was only used temporarily intraoperatively to aid reduction, there was no increased risk of complications or a poorer outcome. The pin used in this scenario is most likely smaller and, thus, less likely to damage important intra-articular structures. If the surgeon decides to leave a temporary pin in situ to aid postoperative immobilisation, its small size is more likely to result in complications such as breakage. In order to avoid such complications, any temporary pins are recommended to be removed at the end of surgery.

Postoperative immobilisation was used in 56.9% patients. Contrary to our hypothesis, a lack of postoperative immobilisation was not related to poorer outcome. An experimental study in dogs showed that early mobilisation after surgical repair of multiple stifle ligaments does not compromise ligament healing or result in undue ligament laxity. In fact, the mobilised stifles were found to be more stable and the medial collateral ligaments stronger.^21^ Surprisingly, in our study, postoperative immobilisation was not statistically associated with complications overall. We speculated that this may be due to the low number of cats and the high number of variables, including different techniques and varying durations of immobilisation. However, considering the severity of complications postoperative immobilisation may cause (eg, tibial or femoral fracture, pin breakage or loosening), along with the fact that a lack of postoperative immobilisation was not associated with a poorer outcome, surgeons must carefully consider the value of this intervention.

Postoperative patellar luxation was seen in five cats and has not previously been reported in cats with disrupted stifle joints. In 3/5 cats the patellar luxation was addressed surgically. None of the cats was reported to have patellar luxation preoperatively; therefore, it is likely to be secondary to the severe soft tissue damage seen with this injury, or to poor surgical technique leading to inadequate reduction or limb alignment. It is essential that the surgeon carefully assesses patellar mobility once the ligament repairs are completed. Meticulous repair of the joint capsule and surrounding soft tissues should be performed, if possible, to reduce the likelihood of this complication developing. In a study conducted in dogs, where patellar luxation occurred as a complication of surgical intervention for cranial cruciate ligament rupture, the authors speculated that persistent craniocaudal instability with possible subsequent patellar instability may have contributed to patellar luxation.^22^ A similar speculation can be made about the cats in our study.

The rate of STCs in our study was high (62.5%), as was the number of cats requiring revision surgery (23.9%). LTCs occurred in 17.5% of cats, with the most common complication being persistent lameness. This is most likely due to the severity of the injury and the development of osteoarthritis, or persistent instability of the joint. Therefore, the owners of cats with multiple stifle ligament injuries should be informed about the likelihood of complications that may require a second surgery, and the possibility of persistent lameness, despite surgical treatment.

The FMPI is a valuable clinical metrology instrument and was used to obtain long-term follow-up information; however, only eight questionnaires were available for analysis. Owing to the low number of questionnaires received, the FMPI score was not used for final outcome analysis; however, the results provide long-term follow-up in cats with disrupted stifles (mean 5.6 years), which has not been previously reported. Many cats were deceased at the time of the study, many owners’ contact details were not valid or available, and not all owners responded to the questionnaire. Of those who responded, half of the cats had a maximum score, indicating no functional deficits. This shows that a good outcome can be maintained in the long term and surgical management of traumatic stifle injury does not necessarily lead to limited function. However, owing to the low number of questionnaires returned, these results must be interpreted carefully. The results also showed that patients can take several months to recover fully. In two of the patients that had a maximum FMPI score, the outcomes from the veterinary records were scored as fair and good, respectively. This could indicate a prolonged recovery time in cats with multi-ligament injuries of the stifle vs cats suffering from other injuries. Additionally, a caregiver placebo effect has been showed to be common in the evaluation of patient response to treatment for osteoarthritis;^23^ therefore, this effect should be considered when interpreting owners’ reports. Conversely, two patients who had excellent outcomes based on the veterinary records had lower FMPI scores than anticipated. At the time of the questionnaire, both of these cats were elderly, with one questionnaire completed 6 years postoperatively and one 12 years postoperatively. Elderly cats often suffer from primary degenerative joint disease,^24^ which is likely to have contributed to the low FMPI score.

### Limitations

The limitations of this study include its retrospective nature and that the data were collected by multiple authors. Patients lost to follow-up may have experienced complications not reported in the record. Differences in record collection, cat management or execution of the surgical procedure between institutions may also have contributed to inadvertent omission of data.

The duration of postoperative treatment with anti-inflammatory drugs was not recorded, and neither was whether the patients were receiving medication at the time of their last follow-up. This represents a limitation of our study as the administration of some medications may have altered the outcome recorded. Additionally, owing to the large number of cats the follow-up period was highly variable; this variability led to cats with a wide range of follow-up times being directly compared. Furthermore, the outcome lacks objective patient outcome measures.

## Conclusions

Surgical treatment of traumatic stifle injury in cats is associated with a high rate of STCs, with almost a quarter of cats requiring revision surgery. Medial meniscal injury, revision surgery and the use of a transarticular pin postoperatively are associated with a poorer outcome. The latter was also associated with a higher complication rate. Postoperative immobilisation had no positive effect on outcome and may therefore not be required. Despite the high complication rate, the overall outcome was good to excellent in 62.3% of cats (n = 38/61), and good limb function can be maintained in the long term, despite the degenerative joint disease.
